# Effects of cognitive behavioral therapy on anxiety and depression in patients with myocardial infarction: a systematic review and meta-analysis

**DOI:** 10.3389/fpsyg.2026.1713464

**Published:** 2026-02-17

**Authors:** Xin Wei, Lin Fu, Huiling Liu, Zhihong Huang, Xiaoqian Lu

**Affiliations:** 1 Precision Treatment Center for Coronary Heart Disease, Zhongshan People’s Hospital, Zhongshan, Guangdong, China; 2 School of Nursing and Health, Henan University, Kaifeng, Henan, China

**Keywords:** anxiety, cognitive behavioral therapy, depression, meta-analysis, myocardial infarction, sleep quality

## Abstract

**Background:**

Anxiety and depressive symptoms are highly prevalent comorbidities among patients with myocardial infarction (MI). Although cognitive behavioral therapy (CBT) is a well-established intervention for depression, its efficacy in MI patients remains inconclusive.

**Objective:**

To evaluate the effects of CBT on anxiety, depressive symptoms, and sleep quality in patients following MI.

**Design:**

This is a systematic review and meta-analysis. The study followed the PRISMA 2020 guidelines for reporting.

**Methods:**

Nine electronic databases were searched from inception to March 2025 to identify randomized controlled trials (RCTs) evaluating CBT in patients with MI. Two independent researchers screened the literature, assessed study quality, and extracted data based on predefined inclusion and exclusion criteria. Random-effects meta-analyses were performed to calculate mean differences, with statistical analyses conducted using Stata 15.0.

**Results:**

Eleven RCTs involving 1,575 participants were included. The findings showed that CBT led to greater improvements in anxiety and depressive symptoms compared with control interventions. In addition, CBT significantly improved sleep quality among patients after MI.

**Conclusion:**

CBT is associated with improvements in psychological and sleep outcomes following MI. However, the existing evidence shows high variability and heterogeneity. Further large-scale, high-quality trials are needed to confirm these findings and develop standardized protocols for implementing CBT in this patient population.

**Systematic review registration:**

https://www.crd.york.ac.uk/prospero/display_record.php?ID=CRD42025636352, identifier CRD42025636352.

## 1 Introduction

Myocardial infarction (MI) remains a leading cause of global morbidity and mortality. While advances in revascularization have improved survival, a significant majority of post-MI patients experience clinically relevant anxiety and depression, which are themselves independent risk factors for adverse cardiovascular outcomes and increased mortality (Santos, 2018; Serpytis et al., 2018; Roth et al., 2020; Alpert, 2023). These psychological comorbidities, along with frequently impaired sleep quality, substantially hinder recovery and long-term prognosis (van Melle et al., 2004; Roest et al., 2010; Meijer et al., 2011; Andrechuk and Ceolim, 2016).

The management of post-MI psychological distress is clinically challenging. Although pharmacotherapy is common, concerns about potential cardiovascular side effects persist (Benazon et al., 2005; Fehr et al., 2019). CBT offers a promising non-pharmacological alternative by targeting maladaptive thoughts and behaviors (Zhang et al., 2022). While previous meta-analyses support the efficacy of psychological interventions, including CBT, for broader coronary heart disease populations (Li Y.-N. et al., 2021; Nuraeni et al., 2023), their specific and pooled effects exclusively for MI patients remain inadequately synthesized. Existing evidence is contradictory, with some trials reporting significant benefits of CBT (Zeighami et al., 2018; Nourisaeed et al., 2021; Johnsson et al., 2025) and others showing limited effects (Norlund et al., 2018). Moreover, no meta-analysis has specifically integrated the evidence on CBT’s impact on sleep quality in this population.

To address these gaps, this systematic review and meta-analysis aims to: (1) Quantify the efficacy of CBT in reducing anxiety and depressive symptoms in patients with MI. (2) Evaluate the effect of CBT on sleep quality in this population. (3) Explore potential moderators of treatment efficacy, such as intervention duration. The findings will provide crucial evidence to guide clinical decision-making and optimize psychological interventions in cardiac rehabilitation programs, ultimately improving patient outcomes and quality of life.

## 2 Materials and methods

This systematic review was registered in PROSPERO (CRD42025636352) and has been conducted and reported following the Preferred Reporting Items for Systematic Reviews and Meta-Analyses (PRISMA) 2020 guidelines (Page et al., 2021).

### 2.1 Eligible criteria

The selection criteria were established based on the Population, Intervention, Comparison, Outcomes, and Study design (PICOS) framework. Studies were considered eligible if they met the following criteria: (1) inclusion of patients with MI; (2) incorporation of interventions using CBT or CBT-based approaches, such as mindfulness techniques, cognitive restructuring, and behavioral activation, within the cognitive behavioral therapeutic framework; (3) inclusion of a control group receiving routine care (UC), control care (CC), or other active therapies; (4) reporting of at least one of the following outcomes: anxiety, depression, or sleep quality; and (5) RCTs published in English or Chinese.

Studies involving qualitative studies, animal studies, *in vitro* studies, observational studies, reviews, letters, conference papers, and study protocols or studies without full-text availability were excluded.

### 2.2 Search strategies and study selection

Inclusive literature was searched in PubMed, Web of Science, Embase, Cochrane Library, PsycINFO, CINAHL, CNKI, WanFang, and Chinese Biomedical (CBM) databases, from the date of establishment to March 28, 2025. The search terms were developed focused on the keywords “Myocardial Infarction” AND “Cognitive Behavioral Therapy” (Supplementary Appendix 1). Additionally, reference lists of the finalized articles and relevant systematic reviews were manually reviewed to identify any additional eligible studies.

The study selection process followed a pre-defined, multi-stage sampling procedure: (1) De-duplication: Records from all databases were imported into EndNote X9 for duplicate removal. (2) Title/Abstract Screening: Two reviewers independently screened titles and abstracts against eligibility criteria using Rayyan software. (3) Full-Text Review: Potentially eligible articles were retrieved and independently assessed in full text by the same two reviewers. (4) Consensus and Adjudication: Disagreements at any stage were resolved through discussion. Persistent disagreements were arbitrated by a third senior researcher. This multi-reviewer process constitutes a form of investigator triangulation to minimize selection bias.

### 2.3 Data extraction and management

A pilot-tested, standardized data extraction form was developed in Microsoft Excel. Data extraction was performed in duplicate by two independent reviewers to ensure accuracy (methodological triangulation). The form captured: (1) study identification details (first author name, publication year, geographic location), (2) participant demographics (sample size, average age, sex composition), (3) intervention characteristics (type, frequency, duration), (4) outcome measures (specific indicators), and (5) supplementary methodological information. Discrepancies in extracted data were reconciled by referring back to the original article and consensus discussion.

### 2.4 Risk of bias and certainty of evidence assessment

Risk of Bias: The methodological quality of the included studies was evaluated using the Cochrane Risk of Bias Assessment Tool (ROB 2.0) (Sterne et al., 2019). This tool was selected for its validation, specificity to RCTs, and detailed domain-based assessment. This comprehensive tool systematically assesses potential bias across five critical domains: (1) randomization process, (2) deviations from intended interventions, (3) missing outcome data, (4) measurement of outcomes, and (5) selection of reported results. Two reviewers independently applied ROB 2.0, judging each domain as “low,” “some concerns,” or “high” risk. Disagreements were resolved as above.

Certainty of Evidence: The quality of evidence for each outcome was assessed using the online version of GRADEpro GDT software (Mendoza et al., 2017). The evaluation was conducted independently by two reviewers. RCTs were initially assumed to provide high-quality evidence, and the certainty of evidence for each outcome was then rated as high, moderate, low, or very low based on five downgrading domains: risk of bias, inconsistency, indirectness, imprecision, and publication bias.

### 2.5 Data analysis

The analysis employed standard mean differences (SMD) with corresponding 95% confidence intervals (CI) to synthesize the data. Effect sizes from individual studies were visually represented using forest plots. Heterogeneity was evaluated using Cochran’s Q test and quantified with the *I*^2^ statistic, where values exceeding 50% were considered to indicate substantial heterogeneity. For studies with *I*^2^ ≤ 50%, a fixed-effect model was applied; otherwise, a random-effects model was utilized. Publication bias was assessed for outcomes with 10 or more studies through funnel plot examination and Egger’s test. All statistical analyses were performed using Stata version 15.0, with a significance level set at α = 0.05.

To explore potential sources of heterogeneity, sensitivity analyses were conducted by sequentially removing individual studies and recalculating the overall effect size. Additionally, subgroup analyses were employed to examine the effectiveness of CBT across different delivery methods. These methods included variations in intervention duration, delivery format (internet-based or telephone-based, or face-to-face), and therapy type (traditional CBT vs. third-generation CBT) in addressing anxiety, depression, and sleep quality among MI patients.

When multiple reports were identified from the same study population, we combined them into a single study entry to avoid duplication of participants. For example, Norlund et al. (2018) (Norlund et al., 2018) and Humphries et al. (2021) (Humphries et al., 2021) both described outcomes from the same cohort and were thus treated as one study in the analysis.

## 3 Results

### 3.1 Literature search results

**FIGURE 1:**
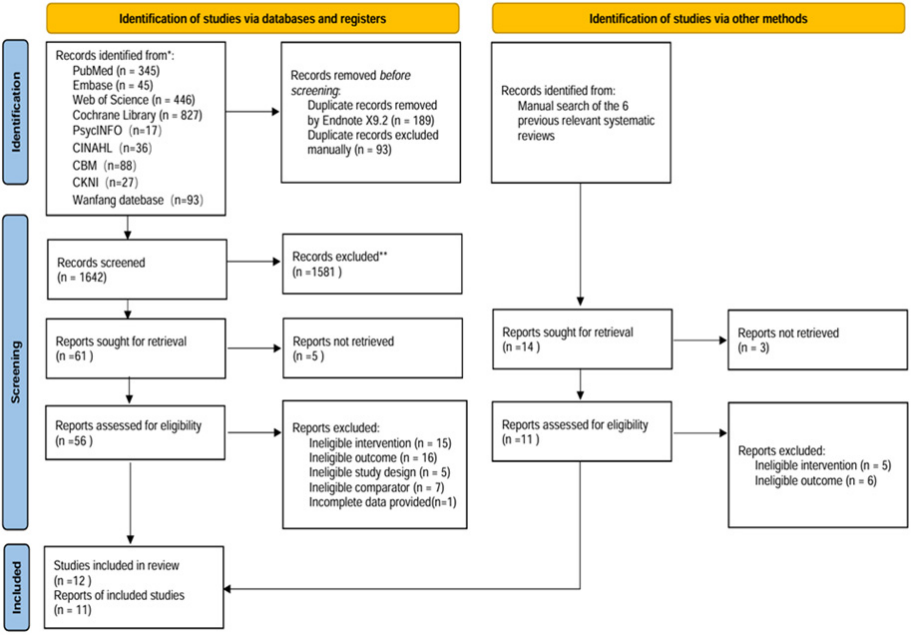
Flowchart of the literature search strategy.

The systematic search across nine databases initially identified 1,924 potentially relevant articles. Following the removal of 282 duplicate records, 1,586 articles were excluded based on title and abstract screening. Subsequent full-text review led to the exclusion of 44 additional articles that did not meet the inclusion criteria. In addition, 14 potentially relevant studies were identified through manual searching of previous relevant systematic reviews, but all were excluded after full-text screening as they did not meet the inclusion criteria. Ultimately, 12 articles reporting on 11 distinct studies were selected for inclusion in the meta-analysis. The detailed flow of the literature screening process is illustrated in Figure 1.

### 3.2 Basic characteristics of the included literature

The systematic review included 11 studies published between 1993 and 2025, encompassing a total of 1,575 MI patients. The psychological interventions implemented in the included studies encompassed a range of therapeutic approaches: CBT (Wang et al., 2011; Ghiasi et al., 2018; Norlund et al., 2018; Humphries et al., 2021; Ning and Wei, 2023; Li et al., 2024), behavioral therapy (BT) (Brown et al., 1993), rational emotive behavioral therapy (Wang et al., 2022), cognitive behavioral stress management (Chen et al., 2024), mindfulness-based cognitive therapy (MBCT) (Spruill et al., 2025), and mediated positive stress reduction (MBSR) (Liang et al., 2019; Wu et al., 2023). For intervention format, 2 studies were conducted online or by telephone (Norlund et al., 2018; Humphries et al., 2021; Spruill et al., 2025), while nine studies were delivered in a face-to-face format (Brown et al., 1993; Wang et al., 2011; Ghiasi et al., 2018; Liang et al., 2019; Wang et al., 2022; Ning and Wei, 2023; Wu et al., 2023; Chen et al., 2024; Li et al., 2024). The mean intervention duration was 7 weeks, with six trials having an intervention duration of less than 7 weeks (Wang et al., 2011; Liang et al., 2019; Wang et al., 2022; Ning and Wei, 2023; Wu et al., 2023; Li et al., 2024). The median study mean age was 59.72 years, with an interquartile range of 57.94–60.84. Detailed information on the characteristics of the included studies is shown in Table 1.

**TABLE 1:** Detailed information about the included literature.

Author year	Country	Characteristics of study participants (EG/CG)			Interventions		Duration of the intervention	Forms of the intervention	Outcome indicator
		Sample size	Age (mean ± SD)	Gender (male : female)	Experimental group	Control group			
Norlund et al. (2018)	Swedish	117/122	58.4 ± 9.0/60.8 ± 7.8	73:44/86:36	CBT	UC	14 weeks	Online	HADS-A HADS-D
Humphries et al. (2021)	Swedish	117/122	58.4 ± 9.0/60.8 ± 7.8	73:44/86:36	CBT	UC	14 weeks	Online	HADS-A HADS-D
Chen et al. (2024)	China	125/125	62.8 ± 10.2/63.6 ± 9.7	87:38/95:30	CBSM	CC	12 weeks	Face to face	HADS-A HADS-D
Wu et al. (2023)	China	50/50	59.72 ± 6.43/60.42 ± 7.12	37:13/35:15	MBSR + CR	CR	5–7 days	Face to face	SAS SDS
Wang et al. (2022)	China	86/86	62.10 ± 6.39/61.32 ± 6.66	52:34/55:31	REBT	UC	3 weeks	Face to face	SAS SDS
Wang et al. (2011)	China	91/90	62 (all)	102:79 (all)	CBT	UC	During hospitalization	Face to face	PSQI
Ning and Wei (2023)	China	55/55	56.44 ± 7.24/56.98 ± 2.33	Unknown	CBT	UC	28 days	Face to face	HADS-A HADS-D PSQI
Brown et al. (1993)	America	20/20	63.55 ± 7.43/57.65 ± 7.82	11:9/18:2	BT	UC	12 weeks	Face to face	BDI
Spruill et al. (2025)	America	67/63	60.2 ± 12.2/59.4 ± 13.5	All female	MBCT	UC	8 weeks	Online (by telephone)	HADS-A PHQ-9
Ghiasi et al. (2018)	Iran	15/15	56.60 ± 5.82 (all)	9:6/7:8	CBT	UC	8 weeks	Face to face	GHQ-28
Liang et al. (2019)	China	58/58	55.12 ± 6.17/55.41 ± 6.25	33:25/32:26	MBSR	UC	7 days	Face to face	SAS SDS PSQI
Li et al. (2024)	China	70/78	58.3 ± 9.3/59.9 ± 9.5	60:10/67:11	VR-CBT	Standard mental health support	7 days	Face to face	HAMA PHQ-15 PSQI

### 3.3 Risk of bias assessment of the included literature

**FIGURE 2:**
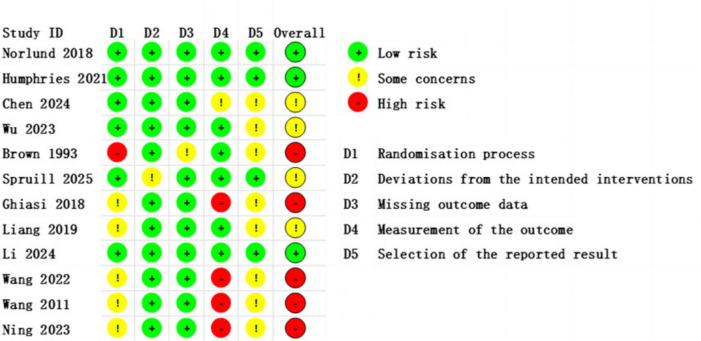
Risk of Bias Assessment traffic light plot, showing the evaluation results of individual studies across different domains.

**FIGURE 3:**
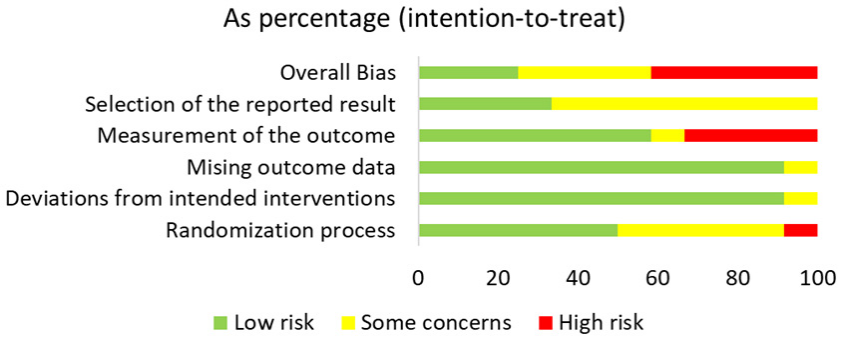
Bar chart of overall distribution across bias domains, displaying the percentage of studies rated as low risk, some concerns, or high risk for each predefined domain (D1–D5).

This study assessed the risk of bias in the included studies using the ROB 2 tool (Supplementary Appendix 1). The results indicated that most studies were judged to have a low or some concerns regarding risk of bias, while only a few exhibited some or high risk of bias, particularly in the domains of blinding implementation and outcome measurement. These methodological limitations may affect the robustness of the overall conclusions. A detailed summary of the risk of bias across all domains for each study is presented in Figures 2, 3.

### 3.4 Results of the meta-analysis

#### 3.4.1 Primary outcomes

##### 3.4.1.1 Anxiety

**FIGURE 4:**
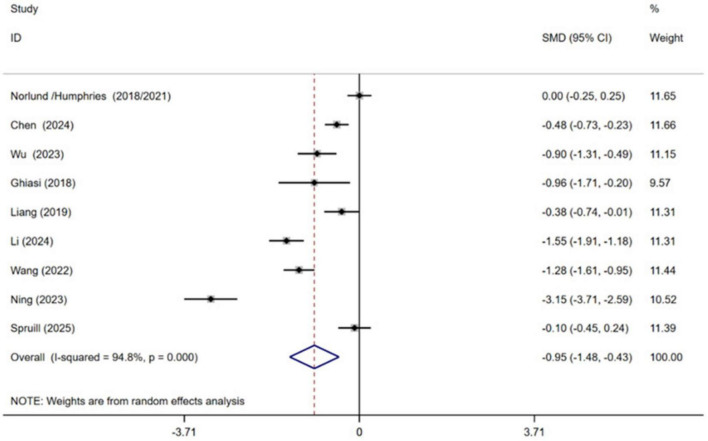
Meta-analysis results for anxiety.

Nine independent RCTs of the included trials investigated the efficacy of CBT in alleviating anxiety among patients with MI, encompassing a total of 1,295 participants (Ghiasi et al., 2018; Norlund et al., 2018; Liang et al., 2019; Wang et al., 2022; Ning and Wei, 2023; Wu et al., 2023; Chen et al., 2024; Li et al., 2024; Spruill et al., 2025). The pooled results demonstrated that CBT yielded a statistically significant improvement in anxiety symptoms with CBT compared to conventional treatment [SMD = −0.95, 95% CI (−1.48, −0.43), *P* < 0.001, *I*^2^ = 94.8%] (Figure 4). However, high heterogeneity between studies was observed.

##### 3.4.1.2 Depression

**FIGURE 5:**
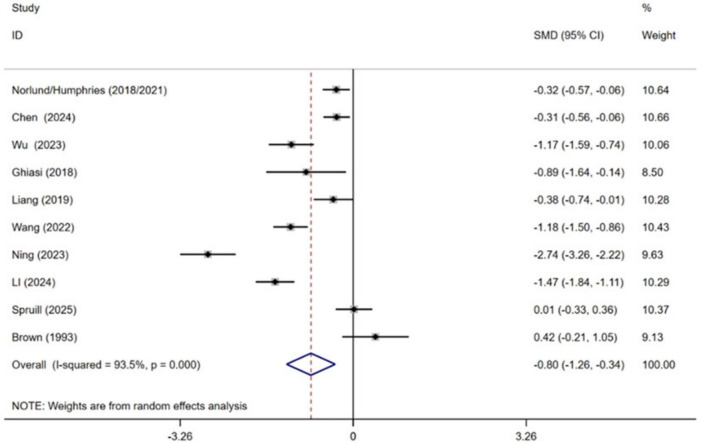
Meta-analysis results for depression.

Ten independent RCTs of the included trials evaluated the effect of cognitive behavioral therapy on managing depression in MI patients, comprising a total of 1,335 participants (Brown et al., 1993; Ghiasi et al., 2018; Norlund et al., 2018; Liang et al., 2019; Humphries et al., 2021; Wang et al., 2022; Ning and Wei, 2023; Wu et al., 2023; Chen et al., 2024; Li et al., 2024; Spruill et al., 2025). Pooled effect sizes indicated that receiving CBT contributed to lower levels of depression in MI patients compared with conventional treatment [SMD = −0.80, 95% CI (−1.26, −0.34), *P* = 0.001, *I*^2^ = 93.5%] (Figure 5). High heterogeneity between studies was observed.

#### 3.4.2 secondary outcomes

##### 3.4.2.1 Quality of sleep

**FIGURE 6:**
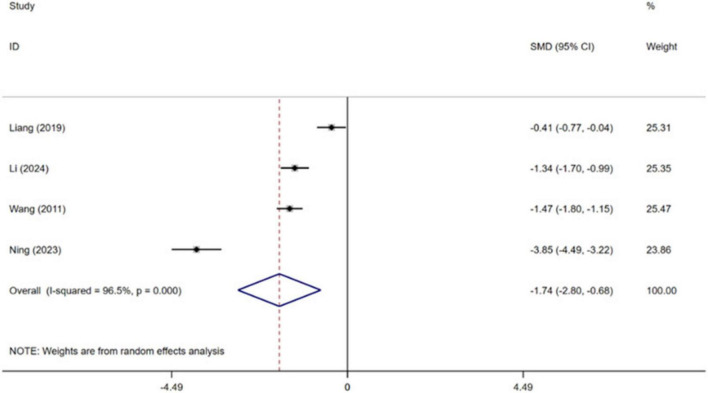
Meta-analysis results for sleep quality.

Four independent RCTs of the included trials evaluated the effect of CBT on sleep quality in patients with MI, encompassing a total of 555 patients (Wang et al., 2011; Liang et al., 2019; Ning and Wei, 2023; Li et al., 2024). Pooled effect sizes indicated that CBT significantly improved patients’ sleep quality compared with conventional treatment [SMD = −1.74, 95% CI (−2.80, −0.68), *P* = 0.001, *I*^2^ = 96.5%] (Figure 6). High heterogeneity was observed between studies.

#### 3.4.3 Subgroup analysis

##### 3.4.3.1 Duration of the intervention

Subgroup analyses were conducted based on intervention duration, categorizing studies into two groups: those with interventions lasting less than or longer than 7 weeks. The results showed that CBT was effective in improving levels of anxiety [SMD = −1.43, 95% CI (−2.17, −0.69), *P* < 0.001, *I*^2^ = 94.4%] and depression [SMD = −1.37, 95% CI (−2.01, −0.72), *P* < 0.001, *I*^2^ = 92.7%] in MI patients receiving shorter-duration interventions (< 7 weeks). In contrast, CBT did not show yield statistically significant improvements in anxiety [SMD = −0.29, 95% CI (−0.62, 0.03), *P* = 0.079, *I*^2^ = 72.7%] and depression [SMD = −0.20, 95% CI (−0.47, 0.06), *P* = 0.130, *I*^2^ = 59.5%] among patients receiving longer-duration interventions (≥ 7 weeks) (details are shown in Table 2).

**TABLE 2:** Results of subgroup analysis of the effect of different intervention durations on patients’ levels of anxiety and depression.

Outcome	Duration of the intervention	No. of studies	Heterogeneity test		Effects model	Meta-analysis results			
			*P*	*I*^2^ (%)		SMD	95% CI	*Z*	*P*
Anxiety	< 7 weeks	5	< 0.001	94.4	Random	−1.43	[−2.17, −0.69]	3.77	< 0.001
	≥ 7 weeks	4	0.012	72.7	Random	−0.29	[−0.62, 0.03]	1.76	0.079
Depression	< 7 weeks	5	< 0.001	92.7	Random	−1.37	[−2.01, −0.72]	4.16	< 0.001
	≥ 7 weeks	5	0.043	59.5	Random	−0.20	[−0.47, 0.06]	1.51	0.130

##### 3.4.3.2 Forms of intervention

Subgroup analyses were conducted according to different forms of intervention (internet or telephone, face-to-face). The results showed that internet-based or telephone-based forms of intervention did not have a positive effect on anxiety [SMD = −0.04, 95% CI (−0.24, 0.17), *P* = 0.728, *I*^2^ = 0.0%] and depression [SMD = −0.17, 95% CI (−0.50, 0.15), *P* = 0.293, *I*^2^ = 56.7%] levels in patients with MI, whereas face-to-face forms of intervention were effective in reducing anxiety [SMD = −1.22, 95% CI (−1.81, −0.64), *P* < 0.001, *I*^2^ = 93.7%] and depression [SMD = −0.97, 95% CI (−1.52, −0.41), *P* = 0.001, *I*^2^ = 93.5%] symptoms in patients (details are shown in Table 3).

**TABLE 3:** Results of subgroup analysis of the effect of different intervention formats on patients’ anxiety and depression levels.

Outcome	Forms of intervention	No. of studies	Heterogeneity test		Effects model	Meta-analysis results			
			*P*	*I*^2^ (%)		SMD	95% CI	*Z*	*P*
Anxiety	Internet or telephone	2	0.637	0.0	Random	−0.04	[−0.24, 0.17]	0.35	0.728
	Face-to-face	7	< 0.001	93.7	Random	−1.22	[−1.81, −0.64]	4.11	< 0.001
Depression	Internet or telephone	2	0.129	56.7	Random	−0.17	[−0.50, 0.15]	1.05	0.293
	Face-to-face	8	< 0.001	93.5	Random	−0.97	[−1.52, −0.41]	3.41	0.001

##### 3.4.3.3 Type of intervention

Subgroup analyses were conducted according to different types of intervention (traditional CBT, third-generation CBT). The results showed that the traditional CBT intervention had a positive effect on both anxiety [SMD = −1.22, 95% CI (−1.97, −0.46), *P* = 0.002, *I*^2^ = 96.3%] and depression [SMD = −0.93, 95% CI (−1.54, −0.31), *P* = 0.003, *I*^2^ = 94.7%] symptoms in patients with MI; the third-generation CBT intervention was effective in reducing patients’ anxiety [SMD = −0.45, 95% CI (−0.89, −0.00), *P* = 0.048, *I*^2^ = 76.5%] levels, while it did not show a statistically significant difference in the effect on patients’ depression [SMD = −0.50, 95% CI (−1.15, 0.15), *P* = 0.134, *I*^2^ = 88.9%] levels; Both types of intervention were effective in improving patients’ sleep quality (details are shown in Table 4).

**TABLE 4:** Results of subgroup analysis of the effects of different intervention types on patients’ anxiety, depression, and sleep quality.

Outcome	Type of intervention	No. of studies	Heterogeneity test		Effects model	Meta-analysis results			
			*P*	*I*^2^ (%)		SMD	95% CI	*Z*	*P*
Anxiety	Traditional CBT	6	< 0.001	96.3	Random	−1.22	[−1.97, −0.46]	3.16	0.002
	Third-generation CBT	3	< 0.001	93.7	Random	−0.45	[−0.89, −0.00]	1.97	0.048
Depression	Traditional CBT	7	< 0.001	94.7	Random	−0.93	[−1.54, −0.31]	2.97	0.003
	Third-generation CBT	3	< 0.001	88.9	Random	−0.50	[−1.15, 0.15]	1.50	0.134
Quality of sleep	Traditional CBT	3	< 0.001	96.0	Random	−2.19	[−3.38, −0.99]	3.59	< 0.001
	Third-generation CBT	1	–	–	–	−0.41	[−0.77, −0.04]	2.16	0.031

#### 3.4.4 Sensitivity analysis

The results of sensitivity analyses suggested that none of the trials would have a disproportionate effect on the overall results, suggesting that the results of the meta-analysis are more robust (Supplementary Appendix 1).

#### 3.4.5 Publication bias

The funnel plot for depression outcomes was approximately symmetric, and Egger’s test indicated no statistically significant publication bias (*P* = 0.341). Taken together, these results suggest that publication bias among the included studies is unlikely to have substantially affected the pooled estimates (Supplementary Appendix 1).

#### 3.4.6 GRADE assessment

The quality of evidence for all outcomes was assessed using the GRADE approach. The results indicated that the certainty of evidence varied across outcomes. The evidence for anxiety and depression was rated as very low, while the evidence for sleep quality was considered low. The main reasons for downgrading were risk of bias, heterogeneity across studies, and indirectness. Detailed GRADE assessments are presented in Supplementary Appendix 1.

## 4 Discussion

Anxiety and depression are prevalent psychological comorbidities in patients with MI (Alexandri et al., 2017). This meta-analysis systematically evaluated 11 studies, including 9 studies focusing on anxiety and 10 studies examining depression. The findings revealed that CBT significantly alleviated both anxiety and depressive symptoms in MI patients. These results corroborate previous meta-analytic findings demonstrating CBT’s efficacy in general cardiovascular populations (Reavell et al., 2018). Notably, the effect sizes observed in this study were comparatively smaller than those reported in non-cardiac populations (Wu et al., 2024). This discrepancy may be attributed to the unique psychological profile of MI patients, potentially influenced by factors such as fear of recurrent infarctions or activity avoidance behaviors.

The duration of CBT for managing anxiety and depressive symptoms in MI patients warrants careful consideration. While Zhang et al.’s meta-analysis suggested that extended intervention durations are necessary for CBT effectiveness (Neher et al., 2019), our findings indicate that short-term CBT interventions (< 7 weeks) demonstrate superior efficacy in reducing both anxiety and depressive symptoms compared to longer-term programs (≥ 7 weeks). This discrepancy may stem from a non-linear relationship between the dose of CBT and the therapeutic response. Evidence indicates that short-term, structured CBT can significantly alleviate patients’ symptoms; however, as the intervention duration increases, the incremental therapeutic benefit may plateau or even diminish (Klein et al., 2024). This phenomenon can be explained by several interrelated factors. First, in extended psychological interventions, patient adherence tends to decline over time—a trend particularly pronounced in the post-MI population, where physical limitations and complex healthcare demands often gradually reduce engagement. For example, a recent trial reported suboptimal adherence rates among MI patients participating in a 14-week CBT program, mainly due to waning treatment motivation (Norlund et al., 2018). Second, patients often experience significant anxiety following the acute phase of MI. Implementing structured CBT early during this stage may enhance the relevance and overall effectiveness of the intervention by aligning with patients’ psychological window of need (Tang et al., 2025). Therefore, the key to treatment lies not simply in extending the duration of therapy, but in ensuring the intensity and adherence during the initial phase, followed by personalized support tailored to individual needs.

The delivery format moderates CBT’s efficacy. Face-to-face delivery was associated with significant improvements in anxiety and depression, whereas internet- based or telephone-based formats showed no significant benefit. This contrasts with evidence supporting digital CBT in broader cardiac populations (Andersson and Cuijpers, 2009; Kwek et al., 2025), a discrepancy potentially explained by the older age profile and associated digital engagement challenges in our MI cohort, as well as the limited number of remote studies analyzed. Future implementation of digital CBT for MI patients should integrate therapist support to address emotional needs and facilitate engagement (Beck, 1963; Andersson and Cuijpers, 2009; Lau et al., 2017).

This study examined CBT interventions that included both traditional CBT, which emphasizes cognitive restructuring and behavioral activation (Kabat-Zinn, 2003), and mindfulness-based CBT (i.e., MBI—a third-wave modality focused on cultivating non-judgmental awareness of present-moment experience) (Li J. et al., 2021). Subgroup analyses indicated that both approaches significantly reduced anxiety levels in MI patients, with traditional CBT potentially offering additional benefit for depressive symptoms, likely due to its direct focus on illness-related cognitions (Abdelaziz et al., 2024). The efficacy of CBT in improving sleep quality was also supported, though evidence remains limited. The considerable heterogeneity observed highlights that CBT should not be regarded as a uniform intervention; future trials should therefore explicitly compare standardized protocols varying in format, dosage, and therapeutic modality.

### 4.1 Strengths and limitations

This study systematically searched and integrated relevant literature from multiple databases, and all included studies underwent rigorous quality assessment, ensuring the reliability of the data and the robustness of the conclusions.

This study has several limitations. First, the relatively small number of included studies, combined with clinical heterogeneity arising from variations in intervention duration, specific protocols, and control conditions across trials, may limit the generalizability of our findings. Second, the assessment of primary outcomes (anxiety and depression levels) relied on measurement tools with inherent subjectivity, which could introduce measurement bias and affect the comparability of results across studies. Third, the included studies were mainly conducted in Sweden, China, the United States, and Iran, with limited evidence from regions such as Africa, South America, and parts of Southeast Asia, which may further constrain the global applicability of the findings. Finally, the substantial heterogeneity observed among the included studies warrants a cautious interpretation of the pooled effect estimates, highlighting the need for further validation through large-scale RCTs.

## 5 Conclusion

This meta-analysis demonstrates that CBT effectively alleviates anxiety, depressive symptoms, and sleep problems in patients with MI. An important and nuanced finding is that the therapeutic benefits of CBT are not uniform but are significantly influenced by intervention characteristics. Specifically, short-term (< 7 weeks), face-to-face, and traditionally structured CBT protocols were associated with the most pronounced improvements, particularly in anxiety and depression.

These findings challenge the conventional view that longer interventions are invariably superior and underscore the importance of optimizing the “dose” and delivery of CBT in this medically vulnerable population. To translate these insights into reliable clinical guidance, future research should move beyond verifying overall efficacy and prioritize large-scale, pragmatic RCTs. Such trials should directly compare optimized short-term versus long-term protocols, evaluate hybrid delivery models to balance accessibility and engagement, and standardize core intervention components and outcome measures to reduce heterogeneity.

For clinical practice, this synthesis suggests that integrating structured, short-term CBT early into cardiac rehabilitation pathways may effectively address acute psychological distress following MI. Ultimately, refining how CBT is delivered can enhance psychological support and overall quality of life for MI survivors, making such care more effective, scalable, and person-centered.

## Data Availability

The original contributions presented in this study are included in this article/Supplementary material, further inquiries can be directed to the corresponding author/s.
