# Correction: Effects of cognitive behavioral therapy on anxiety and depression in patients with myocardial infarction: a systematic review and meta-analysis

**DOI:** 10.3389/fpsyg.2026.1816676

**Published:** 2026-03-11

**Authors:** Xin Wei, Lin Fu, Huiling Liu, Zhihong Huang, Xiaoqian Lu

**Affiliations:** 1 Precision Treatment Center for Coronary Heart Disease, Zhongshan People's Hospital, Zhongshan, Guangdong, China; 2 School of Nursing and Health, Henan University, Kaifeng, Henan, China

**Keywords:** anxiety, cognitive behavioral therapy, depression, meta-analysis, myocardial infarction, sleep quality

Supplemental material [Supplementary Data Sheet 1] was omitted. The file(s) have now been published.

The original version of this article has been updated.

