# Supplementary material for: Effects of cognitive behavioral therapy on anxiety and depression in patients with myocardial infarction: a systematic review and meta-analysis
Source: Front Psychol. 2026 Feb 17;17:1713464. doi: 10.3389/fpsyg.2026.1713464 (PMC12955392; doi:10.3389/fpsyg.2026.1713464)
Supplement: Supplementary file 1 [file Data_Sheet_1.PDF]

Effects of Cognitive Behavioral Therapy on Anxiety and Depression in Patients with Myocardial Infarction: A Systematic Review and Meta-Analysis

Supplementary Contents

Table 1. search strategy

Table 2. Quality assessment of included studies using the Cochrane Risk of Bias tool

Table 3. Summary of GRADE Evidence Profile for Each Outcome

Figure 1. Sensitivity analyses using the method of removing individual studies separately

Figure 2. Funnel Plot for the Depression Outcome

Figure 3. Subgroup Analysis Forest Plot for Anxiety

Figure 4. Subgroup Analysis Forest Plot for Depression

Figure 5. Subgroup Analysis Forest Plot for Sleep Quality

Supplementary Table 1. Search strategy

ID	**PubMed Search strategy**	Hits
#A	"myocardial infarction"[MeSH Terms] OR "myocardial infarct*"[Title/Abstract] OR "heart infarct*"[Title/Abstract]	303958
#B	"cognitive behavioral therapy"[MeSH Terms] OR "CBT"[Title/Abstract] OR "cognit* behavio* therap*"[Title/Abstract] OR "cognit* therap*"[Title/Abstract] OR "cognitive psychotherap*"[Title/Abstract] OR "behavio* therap*"[Title/Abstract] OR "cognit* behavio* intervention"[Title/Abstract]	64597
#C	"Mindfulness-based"[All Fields] AND "cognitive behavioral therapy"[MeSH Terms]) OR "mindfulness based cognitive therap*"[Title/Abstract] OR "MBCT"[Title/Abstract]	3982
#D	"dialectical behavior therapy"[MeSH Terms] OR "dialectical behavior therap*"[Title/Abstract] OR "DBT"[Title/Abstract]	4536
#E	"acceptance and commitment therapy"[MeSH Terms] OR ("Acceptance"[Title/Abstract] AND "commitment therapy"[Title/Abstract]) OR "ACT"[Title/Abstract] OR "acceptance therap*"[Title/Abstract] OR (("accept"[All Fields] OR "acceptabilities"[All Fields] OR "acceptability"[All Fields] OR "acceptable"[All Fields] OR "acceptably"[All Fields] OR "Acceptance"[All Fields] OR "acceptances"[All Fields] OR "acceptation"[All Fields] OR "accepted"[All Fields] OR "accepter"[All Fields] OR "accepters"[All Fields] OR "accepting"[All Fields] OR "accepts"[All Fields]) AND "treatment*"[Title/Abstract]) OR "commitment therap*"[Title/Abstract] OR "commitment treatment*"[Title/Abstract]	510868
#F	#B OR # C OR #D OR #E	572809
#G	"RCT"[Title/Abstract] OR "randomized controlled trial"[Publication Type] OR "controlled clinical trial"[Publication Type] OR "randomized controlled trials as topic"[MeSH Terms] OR "randomized controlled trial"[Publication Type] OR	966443

	"randomized controlled trials as topic"[MeSH Terms] OR "randomized controlled trial"[All Fields] OR "randomised controlled trial"[All Fields]	
#H	#A AND #F AND #G	390
Filter	Language：Chinease and English<br>Species: Humans	345

ID	**Web of Science Search strategy**	Hits
#A	(TI=(Myocardial Infarction OR Myocardial Infarct* OR Heart infarct*)) OR AB=(Myocardial Infarction OR Myocardial Infarct* OR Heart infarct*) and Preprint Citation Index (Exclude – Database)	377706
#B	TI=(Cognitive Behavioral Therapy OR CBT OR cognit* behavio* therap* OR cognit* therap* OR cognitive psychotherap* OR behavio* therap* OR cognit* behavio* intervention OR Mindfulness-based cognitive therap*OR MBCT OR Dialectical behavior therap* OR DBT OR Acceptance and commitment therapy OR ACT OR Acceptance therap* OR Acceptance treatment* OR Commitment therap* OR Commitment treatment*)) OR AB=(Cognitive Behavioral Therapy OR CBT OR cognit* behavio* therap* OR cognit* therap* OR cognitive psychotherap* OR behavio* therap* OR cognit* behavio* intervention OR Mindfulness-based cognitive therap*OR MBCT OR Dialectical behavior therap* OR DBT OR Acceptance and commitment therapy OR ACT OR Acceptance therap* OR Acceptance treatment* OR Commitment therap* OR Commitment treatment*) and Preprint Citation Index (Exclude – Database)	2996681
#C	(TI=(RCT OR randomized controlled trial OR controlled clinical trial OR randomized controlled trials as topic)) OR AB=(RCT OR randomized controlled trial OR controlled clinical trial OR randomized controlled trials as topic) and Preprint Citation Index (Exclude – Database)	716785
#D	#A AND #B AND #C	446

ID	**EMBASE Search strategy**	Hits
#A	('heart infarction'/exp OR 'heart infarction') AND [embase]/lim	451742
#B	('myocardial infarction':ti,ab,kw OR 'myocardial infarct*':ti,ab,kw OR 'heart infarct*':ti,ab,kw) AND [embase]/lim	324957
#C	#A OR #B	492786
#D	('cognitive behavioral therapy'/exp OR 'cognitive behavioral therapy') AND [embase]/lim	40341
#E	('mindfulness-based cognitive therapy'/exp OR 'mindfulness-based cognitive therapy') AND [embase]/lim	1384
#F	('dialectical behavior therapy'/exp OR 'dialectical behavior therapy') AND [embase]/lim	1836
#G	('acceptance and commitment therapy'/exp OR 'acceptance and commitment therapy') AND [embase]/lim	3250

Effects of Cognitive Behavioral Therapy on Anxiety and Depression in Patients with Myocardial Infarction: A Systematic Review and Meta-Analysis

#H	(('cognitive behavioral therapy':ti,ab,kw OR cbt:ti,ab,kw OR 'cognit* behavio* therap*':ti,ab,kw OR 'cognit* therap*':ti,ab,kw OR 'cognitive psychotherap*':ti,ab,kw OR 'behavio* therap*':ti,ab,kw OR 'cognit* behavio* intervention':ti,ab,kw OR 'mindfulness-based cognitive therap*or mbct':ti,ab,kw OR 'dialectical behavior therap*':ti,ab,kw OR dbt:ti,ab,kw OR acceptance:ti,ab,kw) AND 'commitment therapy':ti,ab,kw OR act:ti,ab,kw OR 'acceptance therap*':ti,ab,kw OR 'acceptance treatment*':ti,ab,kw OR 'commitment therap*':ti,ab,kw OR 'commitment treatment*':ti,ab,kw) AND [embase]/lim	345154
#I	#D OR #E OR #F OR #G OR #H	385730
#J	('randomized controlled trial':it OR 'controlled clinical trial':it OR rct:ti,ab,kw OR 'randomized controlled trial':ti,ab,kw OR 'controlled clinical trial':ti,ab,kw OR 'randomized controlled trials as topic':ti,ab,kw) AND [embase]/lim	207276
#K	#C AND #I AND #J	45

ID	Cochrane Library Search strategy	Hits
#A	MeSH descriptor: [Myocardial Infarction] explode all trees	15532
#B	(Myocardial Infarction OR Myocardial Infarct* OR Heart infarct*):ti,ab,kw	42086
#C	#A OR #B	42230
#D	MeSH descriptor: [Cognitive Behavioral Therapy] explode all trees	14291
#E	(CBT):ti,ab,kw OR (cognit* behavio* therap*):ti,ab,kw OR (cognit* therap*):ti,ab,kw OR (cognitive psychotherap*):ti,ab,kw OR (behavio* therap*):ti,ab,kw	109883
#F	(cognit* behavio* intervention):ti,ab,kw OR (Mindfulness-based cognitive therap*):ti,ab,kw OR (MBCT):ti,ab,kw OR (Dialectical behavior therap*):ti,ab,kw OR (DBT):ti,ab,kw	27777
#G	MeSH descriptor: [Mindfulness-Based Cognitive Therapy] explode all trees	0
#H	MeSH descriptor: [Dialectical Behavior Therapy] explode all trees	103
#I	MeSH descriptor: [Acceptance and Commitment Therapy] explode all trees	507
#J	(ACT):ti,ab,kw OR (Acceptance therap*):ti,ab,kw OR (Acceptance treatment*):ti,ab,kw OR (Commitment therap*):ti,ab,kw OR (Commitment treatment*):ti,ab,kw	26586
#K	#D OR #E OR #F OR #G OR #H OR #I OR #J	139998
#L	(randomized controlled trial):pt OR (controlled clinical trial):pt OR (controlled clinical trial):ti,ab,kw OR (randomized controlled trial):ti,ab,kw OR (RCT):ti,ab,kw (Word variations have been searched)	958275
#M	#C AND #K AND #L	847
#N	Trials	827

ID	CINAHL Search strategy	Hits
#A	(TI (Myocardial Infarction OR Myocardial Infarct* OR Heart infarct*)) OR (AB (Myocardial Infarction OR Myocardial Infarct* OR Heart infarct*))	54747

Effects of Cognitive Behavioral Therapy on Anxiety and Depression in Patients with Myocardial Infarction: A Systematic Review and Meta-Analysis

#B	TI (Cognitive Behavioral Therapy OR CBT OR cognit* behavio* therap* OR cognit* therap* OR cognitive psychotherap* OR behavio* therap* OR cognit* behavio* intervention OR Mindfulness-based cognitive therap*OR MBCT OR Dialectical behavior therap* OR DBT OR Acceptance and commitment therapy OR ACT OR Acceptance therap* OR Acceptance treatment* OR Commitment therap* OR Commitment treatment*) OR AB (Cognitive Behavioral Therapy OR CBT OR cognit* behavio* therap* OR cognit* therap* OR cognitive psychotherap* OR behavio* therap* OR cognit* behavio* intervention OR Mindfulness-based cognitive therap*OR MBCT OR Dialectical behavior therap* OR DBT OR Acceptance and commitment therapy OR ACT OR Acceptance therap* OR Acceptance treatment* OR Commitment therap* OR Commitment treatment*)	96496
#C	PT (randomized controlled trial or rct) OR AB (RCT OR randomized controlled trial OR controlled clinical trial OR randomized controlled trials as topic) OR TI (RCT OR randomized controlled trial OR controlled clinical trial OR randomized controlled trials as topic)	256690
#D	#A AND #B AND #C	36

ID	**PsycInfo Search strategy**	Hits
#A	(TI (Myocardial Infarction OR Myocardial Infarct* OR Heart infarct*)) OR (AB (Myocardial Infarction OR Myocardial Infarct* OR Heart infarct*))	4886
#B	(TI (Cognitive Behavioral Therapy OR CBT OR cognit* behavio* therap* OR cognit* therap* OR cognitive psychotherap* OR behavio* therap* OR cognit* behavio* intervention OR Mindfulness-based cognitive therap*OR MBCT OR Dialectical behavior therap* OR DBT OR Acceptance AND commitment therapy OR ACT OR Acceptance therap* OR Acceptance treatment* OR Commitment therap* OR Commitment treatment*)) OR (AB (Cognitive Behavioral Therapy OR CBT OR cognit* behavio* therap* OR cognit* therap* OR cognitive psychotherap* OR behavio* therap* OR cognit* behavio* intervention OR Mindfulness-based cognitive therap*OR MBCT OR Dialectical behavior therap* OR DBT OR Acceptance AND commitment therapy OR ACT OR Acceptance therap* OR Acceptance treatment* OR Commitment therap* OR Commitment treatment*))	200912
#C	(PT (randomized controlled trial OR rct)) OR (TI (RCT OR randomized controlled trial OR controlled clinical trial OR randomized controlled trials "as" topic)) OR (AB (RCT OR randomized controlled trial OR controlled clinical trial OR randomized controlled trials "as" topic))	55850
#D	#A AND #B AND #C	17

ID	**CBM Search strategy**	Hits
#A	"心肌梗死"[常用字段:智能] OR "急性心肌梗死"[常用字段:智能]	147682
#B	"认知行为疗法"[常用字段:智能] OR "认知行为"[常用字段:智能] OR "CBT"[常用字段:智能] OR "正念认知行为"[常用字段:智能] OR "MBCT"[常用字段:智能]	20415

	OR "辩证行为疗法"[常用字段:智能] OR "DBT"[常用字段:智能] OR "接受与承诺疗法"[常用字段:智能] OR "ACT"[常用字段:智能]	
#C	"随机对照"[常用字段:智能] OR "随机"[常用字段:智能] OR "随机分组"[常用字段:智能] OR "RCT"[常用字段:智能]	2039408
#D	#A AND #B AND #C	88

ID	**CNKI Search strategy**	Hits
#A	主题："心肌梗死 + 急性心肌梗死"	/
#B	主题："认知行为疗法 + 认知行为 + 正念认知行为 + 辩证行为疗法 + 接受与承诺疗法"	/
#C	全文："随机对照 + 随机 + 随机分组 + RCT"	
#D	#A AND #B AND #C	39
	期刊	27

ID	**WanFang Search strategy**	Hits
#A	主题："心肌梗死 OR 急性心肌梗死"	/
#B	主题："认知行为疗法 OR 认知行为 0R 正念认知行为 OR 辩证行为疗法 OR 接受与承诺疗法"	/
#C	全文："随机对照 OR 随机 OR 随机分组 OR RCT"	
#D	#A AND #B AND #C	93

Supplementary Table 2. Quality assessment of included studies using the Cochrane Risk of Bias tool (ROB2)

Reference	Bias arising from the randomization process	Bias due to deviations from the intended interventions	Bias due to missing outcome data	Bias in measurement of the outcome	Bias in selection of the reported result	Overall
Norlund, 2018	Low risk	Low risk	Low risk	Low risk	Low risk	Low risk
Humphries, 2021	Low risk	Low risk	Low risk	Low risk	Low risk	Low risk
Chen, 2024	Low risk	Low risk	Low risk	Some concerns	Some concerns	Some concerns
Wu, 2023	Low risk	Low risk	Low risk	Low risk	Some concerns	Some concerns
Brown, 1993	High risk	Low risk	Some concerns	Low risk	Some concerns	High risk

Spruill, 2025	Low risk	Some concerns	Low risk	Low risk	Low risk	Some concerns
Ghiasi, 2018	Some concerns	Low risk	Low risk	High risk	Some concerns	High risk
Liang, 2019	Some concerns	Low risk	Low risk	Low risk	Some concerns	Some concerns
Li, 2024	Low risk	Low risk	Low risk	Low risk	Low risk	Low risk
Wang, 2022	Some concerns	Low risk	Low risk	High risk	Some concerns	High risk
Wang, 2011	Some concerns	Low risk	Low risk	High risk	Some concerns	High risk
Ning, 2023	Some concerns	Low risk	Low risk	High risk	Some concerns	High risk

Supplementary Table 3. Summary of GRADE Evidence Profile for Each Outcome

Certainty assessment							№ of patients		Effect		Certainty	Importance
№ of studies	Study design	Risk of bias	Inconsistency	Indirectness	Imprecision	Other considerations	[CBT Intervention]	[control group]	Relative (95% CI)	Absolute (95% CI)		
**Anxiety**												
9	randomised trials	serious[a]	serious[b]	serious[c]	not serious	none	643	652	-	SMD **0.95 SD lower** (1.48 lower to 0.43 lower)	⨁◯◯◯ Very low[a,b,c]	IMPORTANT
**Depression**												
10	randomised trials	serious[a]	serious[b]	serious[c]	not serious	none	663	672	-	SMD **0.8 SD lower** (1.26 lower to 0.34 lower)	⨁◯◯◯ Very low[a,b,c]	IMPORTANT
**Sleep Quality**												
4	randomised trials	serious[d]	serious[b]	not serious	not serious	none	274	281	-	SMD **1.74 SD lower** (2.8 lower to 0.68 lower)	⨁⨁◯◯ Low[b,d]	IMPORTANT

CI: confidence interval; **SMD:** standardised mean difference

Explanations

a. Some studies were at moderate risk of bias due to unclear randomization methods and potential selection bias.Downgraded

b. Serious inconsistency since I^2 > 50%. Downgraded

c. Different measurement tools were used for the outcome.Downgraded

d. Most studies were rated to have serious risk of bias.Downgraded

Supplementary Figure 1. Sensitivity analyses using the method of removing individual studies separately

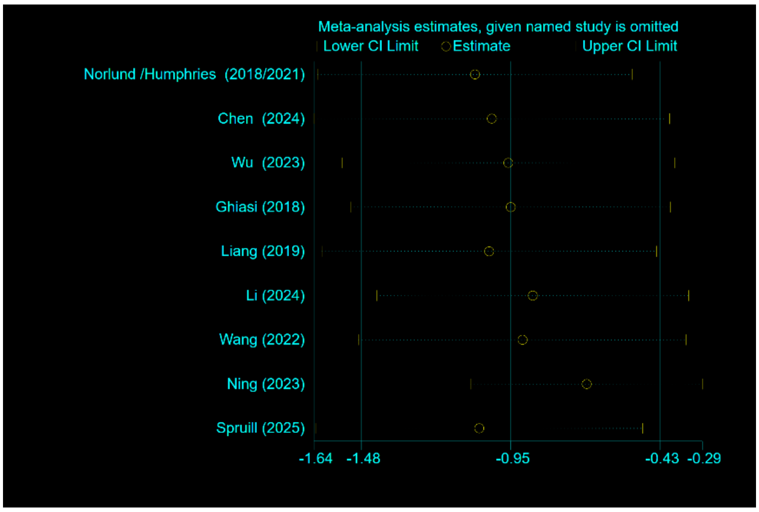

(A) anxiety

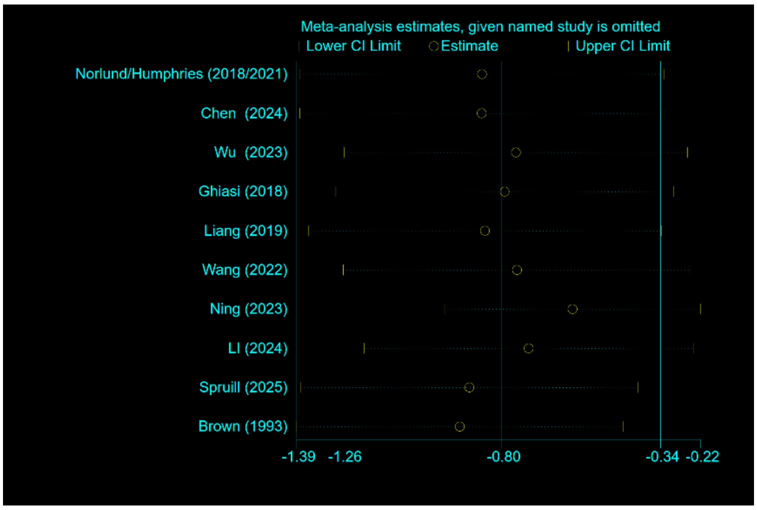

(B) depression

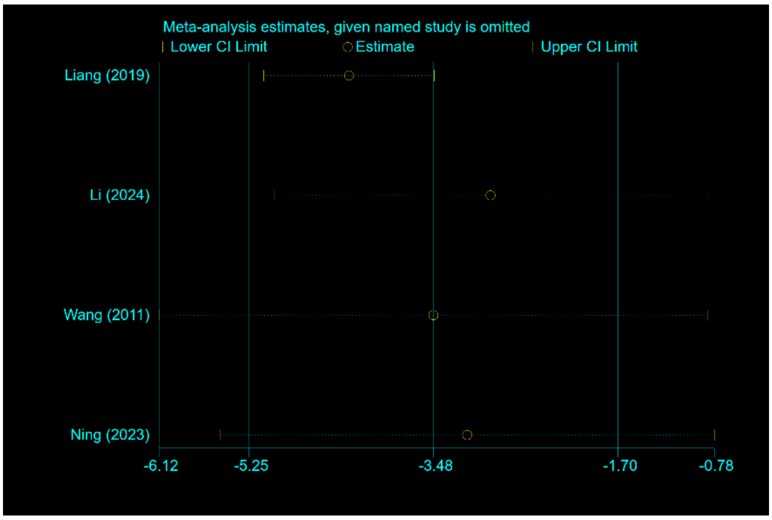

(C) sleep quality

Supplementary Figure 2. Funnel Plot for the Depression Outcome

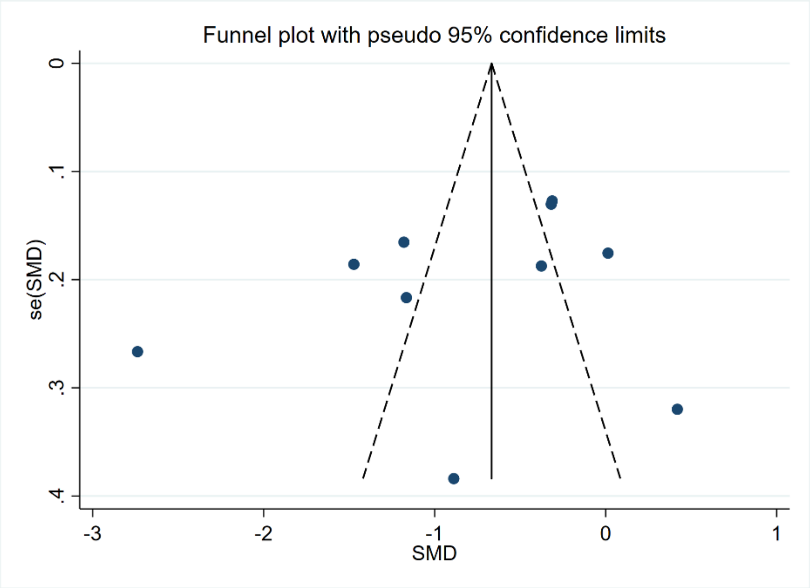

Effects of Cognitive Behavioral Therapy on Anxiety and Depression in Patients with Myocardial Infarction: A Systematic Review and Meta-Analysis

Supplementary Figure 3. Subgroup Analysis Forest Plot for Anxiety

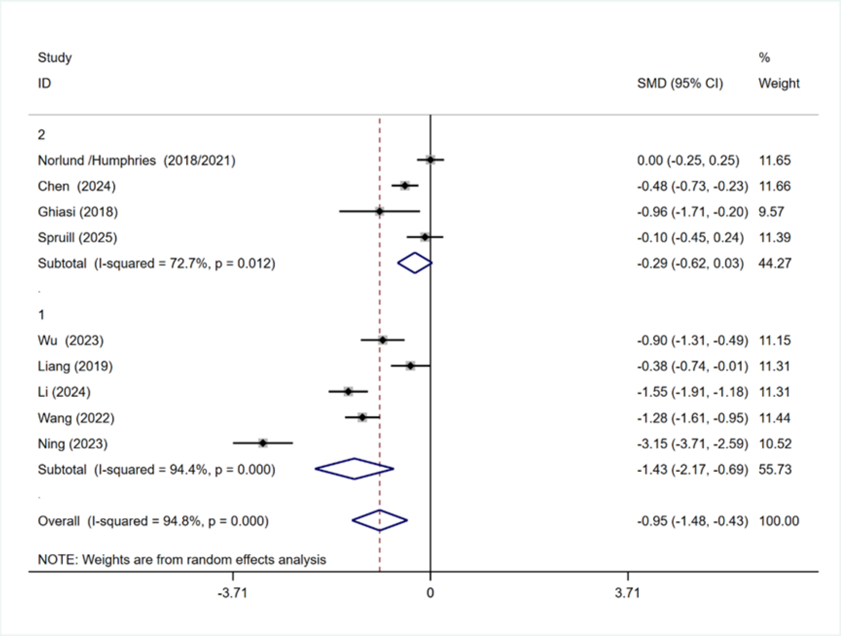

(A) Subgroup Analysis Based on Intervention Duration (<7 weeks vs. ≥7 weeks)

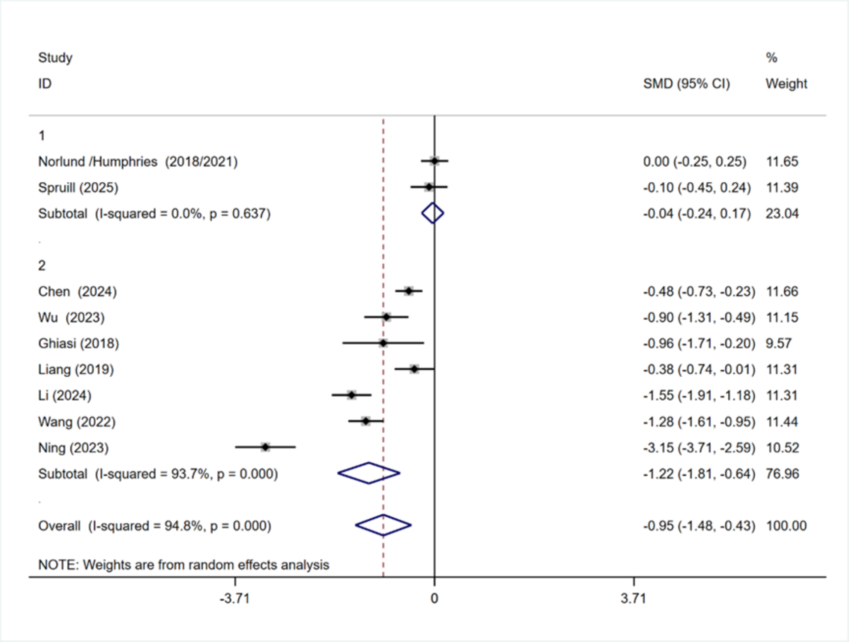

(B) Subgroup Analysis Based on Delivery Mode (Online vs. Offline)

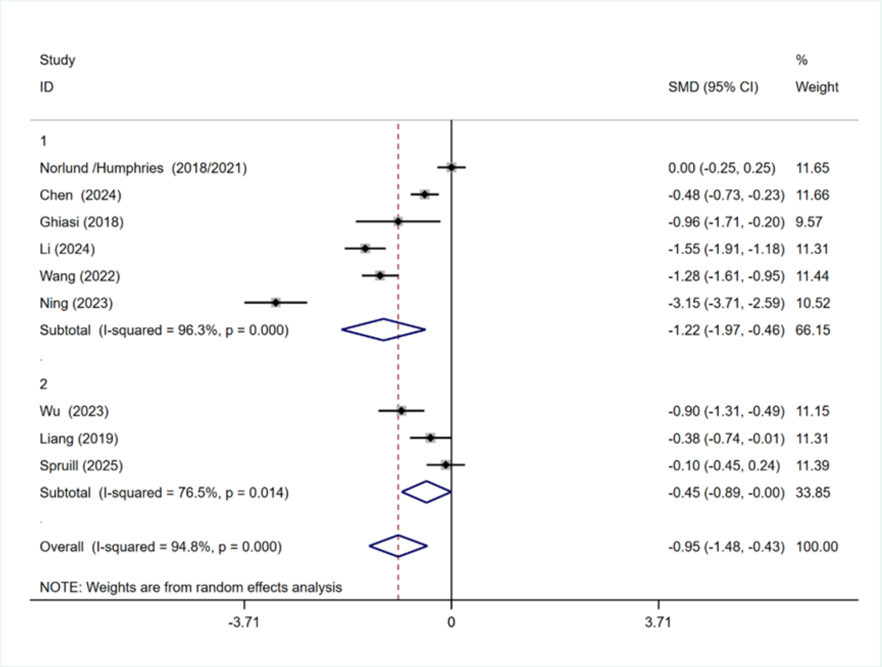

(C) Subgroup Analysis Based on CBT Type (Traditional CBT vs. Third-Wave CBT)

Supplementary Figure 4. Subgroup Analysis Forest Plot for Depression

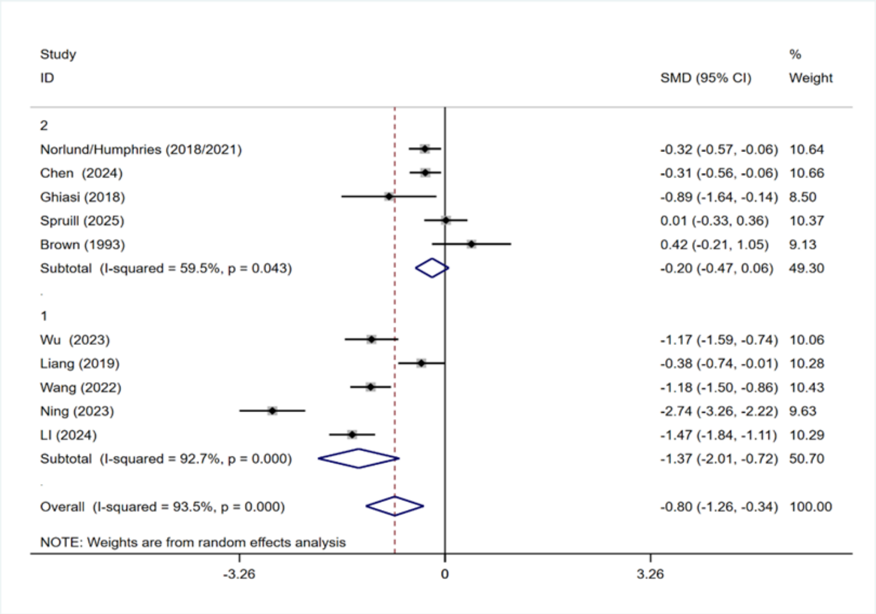

(A) Subgroup Analysis Based on Intervention Duration (<7 weeks vs. ≥7 weeks)

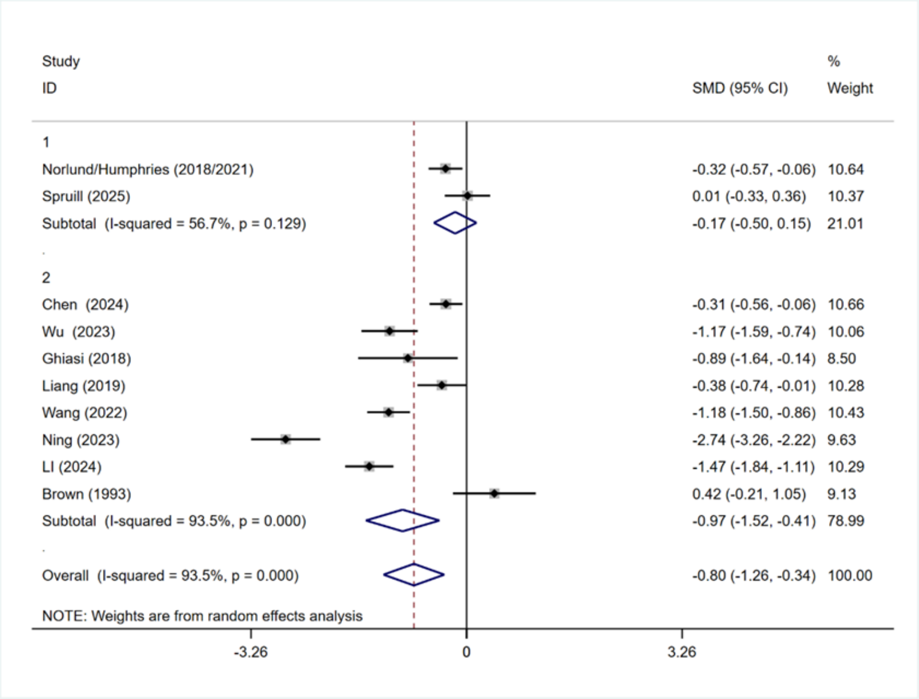

(B) Subgroup Analysis Based on Delivery Mode (Online vs. Offline)

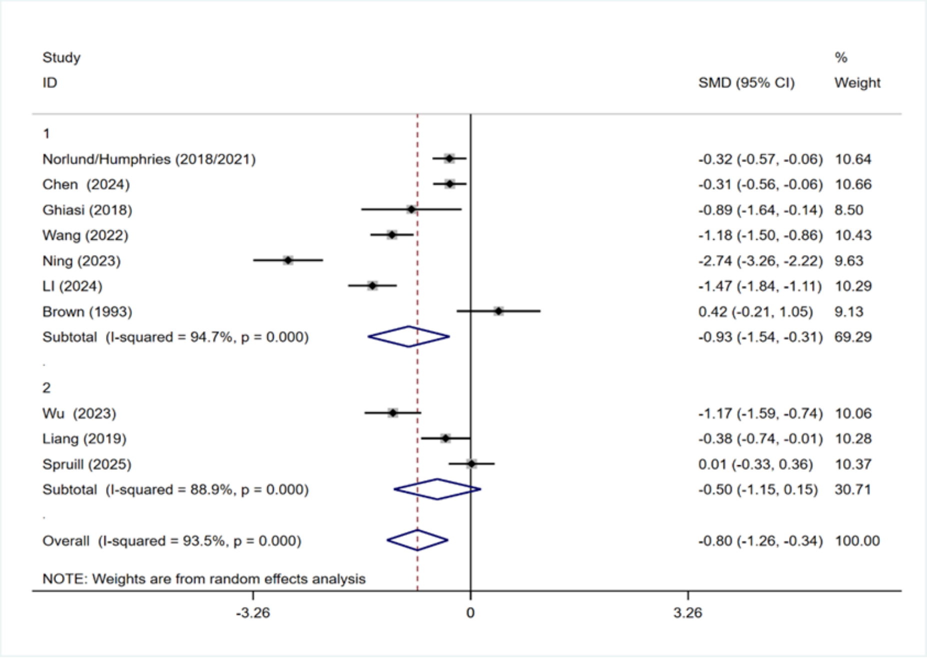

(C) Subgroup Analysis Based on CBT Type (Traditional CBT vs. Third-Wave CBT)

Supplementary Figure 4. Subgroup Analysis Forest Plot for Sleep Quality

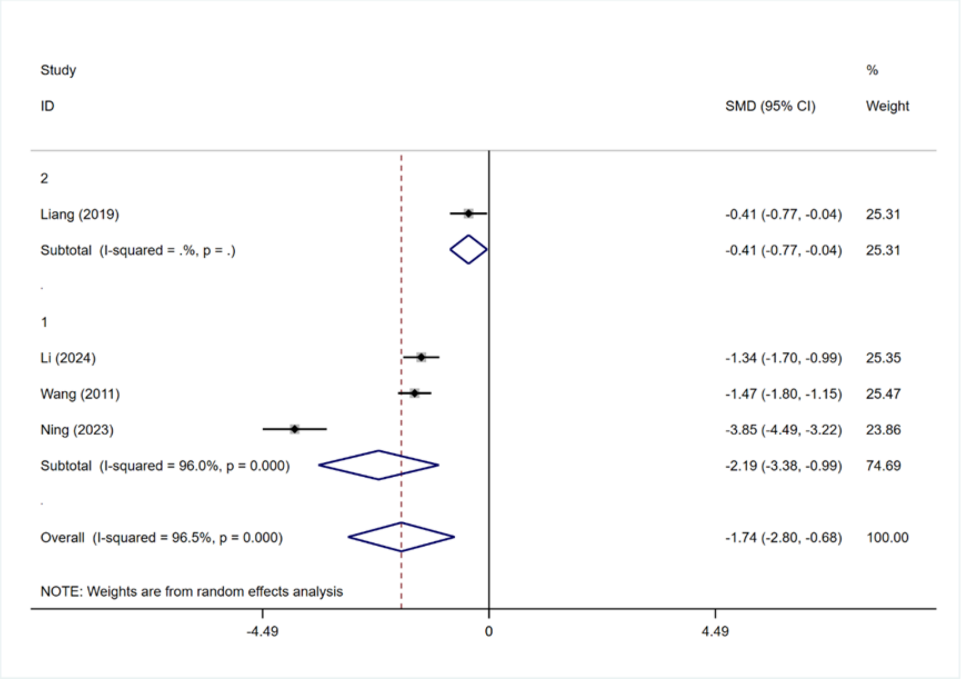

(A) Subgroup Analysis Based on CBT Type (Traditional CBT vs. Third-Wave CBT)
